# Stick-slip nonuniform rotation distortion correction in distal scanning optical coherence tomography catheters

**DOI:** 10.1142/s1793545820500303

**Published:** 2020-11

**Authors:** Jessica Mavadia-Shukla, Jianlin Zhang, Kaiyan Li, Xingde Li

**Affiliations:** Department of Biomedical Engineering, Johns Hopkins University, Baltimore, MD 21205, USA

**Keywords:** Optical coherence tomography, nonuniform rotation distortion correction, endoscope, distal-scanning

## Abstract

We present a robust and fiducial-marker-free algorithm that can identify and correct stick-slip distortion caused by nonuniform rotation (or beam scanning) in distally scanned catheters for endoscopic optical coherence tomography (OCT) images. This algorithm employs spatial frequency analysis to select and remove distortions. We demonstrate the feasibility of this algorithm on images acquired from *ex vivo* rat colon with a distally scanned DC motor-based endoscope. The proposed algorithm can be applied to general endoscopic OCT images for correcting nonuniform rotation distortion.

## 1. Introduction

Nonuniform rotation distortion (NURD) is common in endoscopic OCT imaging. Traditional endoscopic OCT imaging involves mechanical rotation of the entire endoscope at the proximal end through a fiber-optic rotary joint.^1^ The corresponding endoscope must be equipped with a torque applying mechanism in order to transfer rotation from the proximal end to the imaging optics at the distal end. The imaging endoscope/catheter is often encased within a protective transparent plastic sheath, and any resistance in the path between the endoscope and the sheath can cause distortions in the image. This type of distortion is difficult to avoid when imaging through a tortuous pathway where significant bending of the catheter occurs.

Distal scanning endoscopes with miniature micromotors are an alternative for performing circumferential beam scanning that can avoid the aforementioned resistance-based image distortions.^2–9^ Due to size constraints, micromotors incorporated in distal scanning endoscopes often lack feedback mechanisms such as encoders, making it difficult to maintain the rotational velocity. Distal scanning catheters can also exhibit NURD due to an imbalanced load on the motor shaft, or simply due to mechanical instabilities at higher speeds. In certain cases, such as for imbalanced loads on the motor shaft, increasing the torque or rotational velocity can help reduce NURD by overcoming resistance. In the case of mechanical instability at higher speeds approaching to the limit, the only plausible solution may be to decrease the rotational velocity.

However, reducing rotational velocities to prevent NURD is not a viable option as a higher imaging speed is generally preferred and the field has witnessed continued increase in A-scan rates from several hundred kHz^10–12^ to 20.8 million A-scans/sec and beyond.^13^ In order to keep up with ever increasing A-scan rates, miniature micromotors have become a popular choice enabling frame rates to be increased from hundreds^14,15^ to thousands of frames per second^6^ corresponding to hundreds to thousands rotations per second. But as mentioned earlier, one of the drawbacks of either proximal or distal rotation is NURD.

NURD is used as a generic term to describe rotational distortion of which there are mainly two types, (1) translational NURD^16^ and (2) stretch-shrink NURD.^16–18^ The first type of distortion results from axial or lateral displacements of tissue or catheter during imaging. Lateral displacements that move the catheter away from the center of the lumen can cause nonuniform sampling in the radial direction. For a smaller lumen, this does not pose a significant issue in terms of sample uniformity, but can cause distortions in volume rendering of pull-back scans. This has been discussed in detail and is mostly solved by cross-correlation and image registration algorithms.^16,19,20^

Stretch-shrink NURD is caused either by mechanical resistance in the path of a proximal scanning catheter or nonuniform rotational speed of micromotor in a distal scanning catheter, which leads to nonuniform rotational sampling that appears stretched for sparsely sampled regions, or compressed for densely sampled regions. Stretch-shrink NURD can be addressed through resampling and interpolating the image based on fiducial markers.^21^ As a special case, severe stretch-shrink NURD (termed as stick-slip NURD) can occur when the catheter rotation is momentarily halted before enough torque is built up to resume rotation. This stick-slip NURD commonly occurs in distal scanning catheters where the micromotors often cannot afford a large torque or there exists a load imbalance.^14,21^ On OCT images, the resulting distortion manifests as segments of repeated A-lines without any deterministic patterns. Such distortions can lead to an inaccurate representation of imaged tissue structure, making suppression of NURD important to prevent potential clinical misinterpretation of OCT images while enabling high-speed endoscopic imaging. However, as of yet, not many algorithms have been developed to correct images with stick-slip NURD. As we mentioned before, registration-based methods correct displacement distortion and rotational speed variations between frames which are not very suitable for stick-slip NURD.^16,19,20^ A correlation-based image acquisition technique could also identify and remove stick-slip NURDs.^22^ Nevertheless, selecting the right threshold for cross-correlation results is crucial and challenging. This method also demands sufficient oversampling which limits the imaging speed. Another correlation-based method can be used both in low-speed variations and stick-slip NURD correction, but it requires selection of the length of correction window for different measurable speeds and also requires a calibration or a registration algorithm to compensate the resultant nonlinearity of rotational speed between frames.^18,23^

In this paper, we report a robust algorithm for correcting stick-slip NURD. Our method relies on spatial frequency filtering. The spatial filtering method yields a single mask that can be used to correct the whole dataset for a given run (without stopping and restarting the micromotor). This approach does not require any fiducial markers making it easily adaptable to a wide variety of endoscope designs. We will demonstrate the efficacy of the algorithm for endoscopic OCT with *ex vivo* rat colon acquired from a recently developed distal scanning endoscope.

## 2. Methods and Results

The OCT images presented in this paper were obtained with an endoscopic spectral domain OCT (SD-OCT) system. The SD-OCT engine consisted of a home-built broadband mode-locked Ti:Sapphire laser with a central wavelength of 830 nm and 3 dB spectral bandwidth of ~ 150 nm and a customized broadband linear-in-wavenumber spectrometer. The broadband spectrometer employed a 2048-pixel line CCD camera with an A-scan rate up to 70 k A-lines/second. This SD-OCT system incorporated a distal scanning endoscope.^8^

The distal-end scanning endoscope employed a 1.9 mm diameter DC micromotor along with a micro-mirror to perform circumferential beam scanning. The micromotor was capable of rotating up to 300 rotations/second. The effective frame rate was dependent on the sampling requirement of the high-resolution endoscope and the A-scan rate of the line CCD in the spectrometer. The overall endoscope design resembled the one presented in our previous paper,^4^ which had an outer diameter of 3.1 mm and offered an axial resolution of 2.8 *μ*m (in air) and a lateral resolution of 8.7 *μ*m.

Animal model imaging was performed under a protocol approved by the Animal Care and Use Committee at the Johns Hopkins University. For *ex vivo* rat colon imaging, the colon was first flushed with saline before the endoscope was deployed into the colon. Images were acquired at 8.5 frames per second with each frame consisting of 8 k A-lines. The motor was driven independently with a motor driver, and synchronized data acquisition was achieved by matching the motor speed with the imaging frame rate. 3D volumetric datasets were collected by circumferential beam scanning along with pullback.

**Fig. 1.**
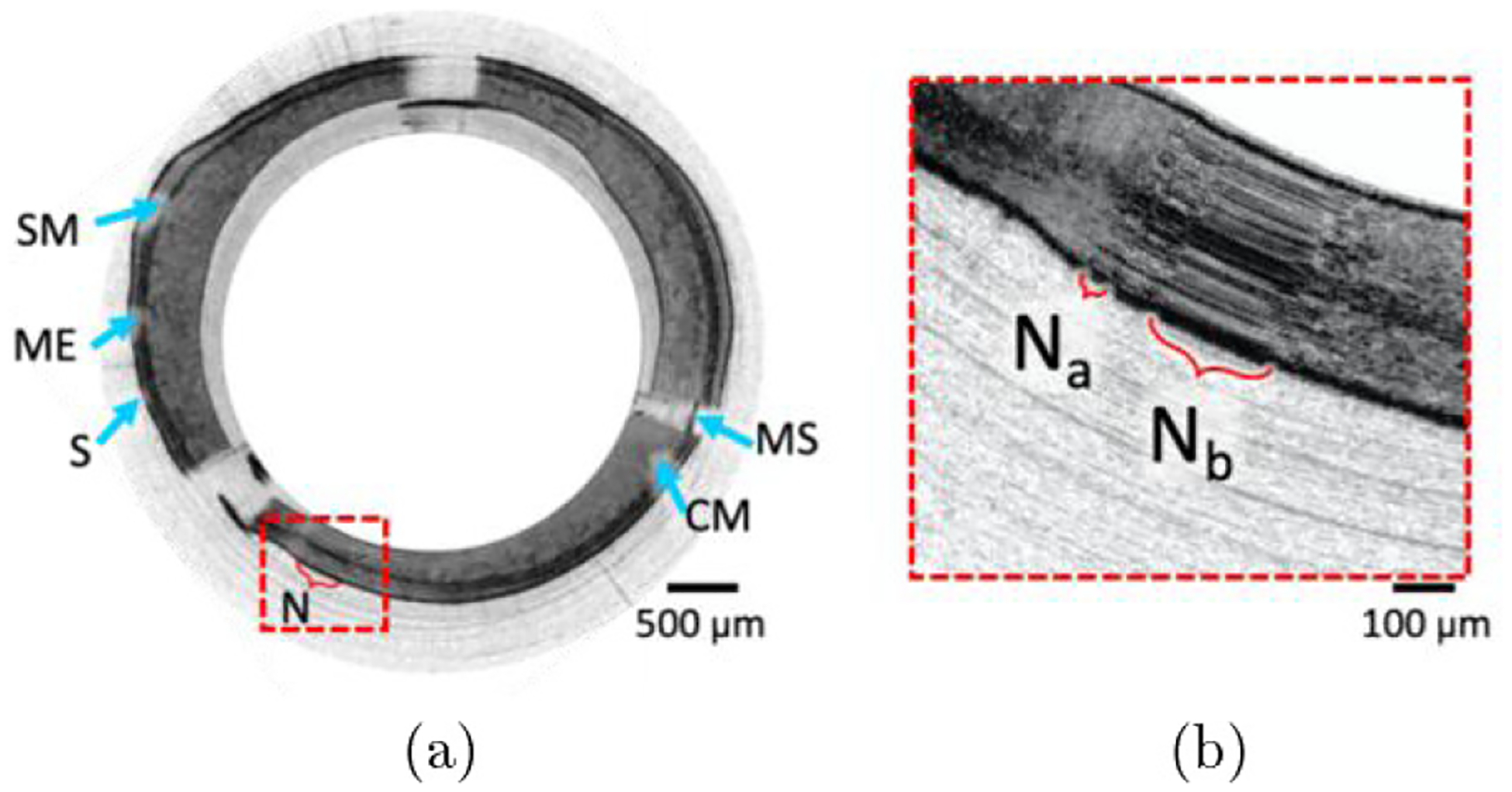
(a) Representative cross-sectional OCT image from *ex vivo* rat colon showing nonuniform rotational distortion labeled with N in the boxed region marked by red dashed line border. The labeled colon wall structures include, CM—colonic mucosa, SM—submucosa, ME—muscularis externa, and S—serosa. MS represents metal strut of distal enclosure. (b) Zoomed-in view of the boxed region in (a), which explicitly shows nonuniformities that have been labeled with *N*_*a*_ and *N*_*b*_ to exemplify two regions of repeated A-lines composing of NURD.

Figure 1(a) shows a representative cross-sectional image where NURD is evident over the region marked by a red bracket. When we zoom into the boxed region, as shown in Fig. 1(b), we can see the NURD region consists of a couple of clusters of repeated A-lines, two of which are denoted *N*_*a*_ and N_b_. The clusters of repeated A-lines in regions *N*_*a*_ and N_*b*_, appear at different locations in the image without any deterministic pattern. While one of these A-lines within the cluster may have unique information about tissue structures, others are redundant and do not represent true tissue structures.

For the purposes of illustrating our NURD removal algorithm, the circumferential OCT endoscopic images are first presented in the unwrapped, rectangular view. It is recognized that the cluster of repeated A-lines have almost identical interferograms meaning, for a given wavenumber *k*, the signal intensity of adjacent interferograms does not vary much versus lateral position *x* in a given image frame. Thus, when the spatial frequency range is examined for a given wavenumber *k* with respect to the spatial location *x*, it can be expected that the cluster of repeated A-lines will reside in the low spatial frequency range. Performing spatial frequency filtering on the raw OCT image data prior to performing the Fourier transform can then filter out NURD regions in the image.

The basic approach for removing stick-slip NURD is to first identify and exclude the unwanted A-lines (i.e., those repeated A-lines). According to the aforementioned analysis, NURD regions are identified in low spatial frequency regions and can be excluded by filtering out low spatial frequencies from the image. However, tissue structures also contain some low spatial frequencies. Consequently, a robust algorithm is required to reliably identify and exclude NURD regions without sacrificing true tissue structures with low spatial frequencies.

Here, a mask-based algorithm is proposed. The first step is to perform high pass filtering (HPF) on the raw SD-OCT data *I*(*k*, *x*) prior to performing the FFT, i.e., *I*_*1*_(*z*, *x*) = *FFT*{*HPF*[*I(k*, *x*)]}. HPF was performed with a Butterworth filter to avoid ripples in the pass-band or stop-band. The HPF cutoff frequency was determined based on the spatial frequency corresponding to the length of the shortest NURD cluster. Given 8192 A-lines per frame and a circumference of 11.6 mm at the focal plane of the endoscope the maximum spatial frequency that could be captured according to Nyquist sampling theorem is 353 mm^−1^.

Figure 1(b) shows two NURD clusters *N*_*a*_ and N_*b*_ where *N*_*a*_ is the shorter NURD cluster. Upon visual inspection of the entire image, *N*_*a*_ has ~ 13 repeated A-lines corresponding to the micromotor being halted for ~ 185 *μ*s and a spatial frequency of 54.3 mm^−1^, i.e., ~ 15% of the spatial frequency range of the raw OCT image data.

**Fig. 2.**
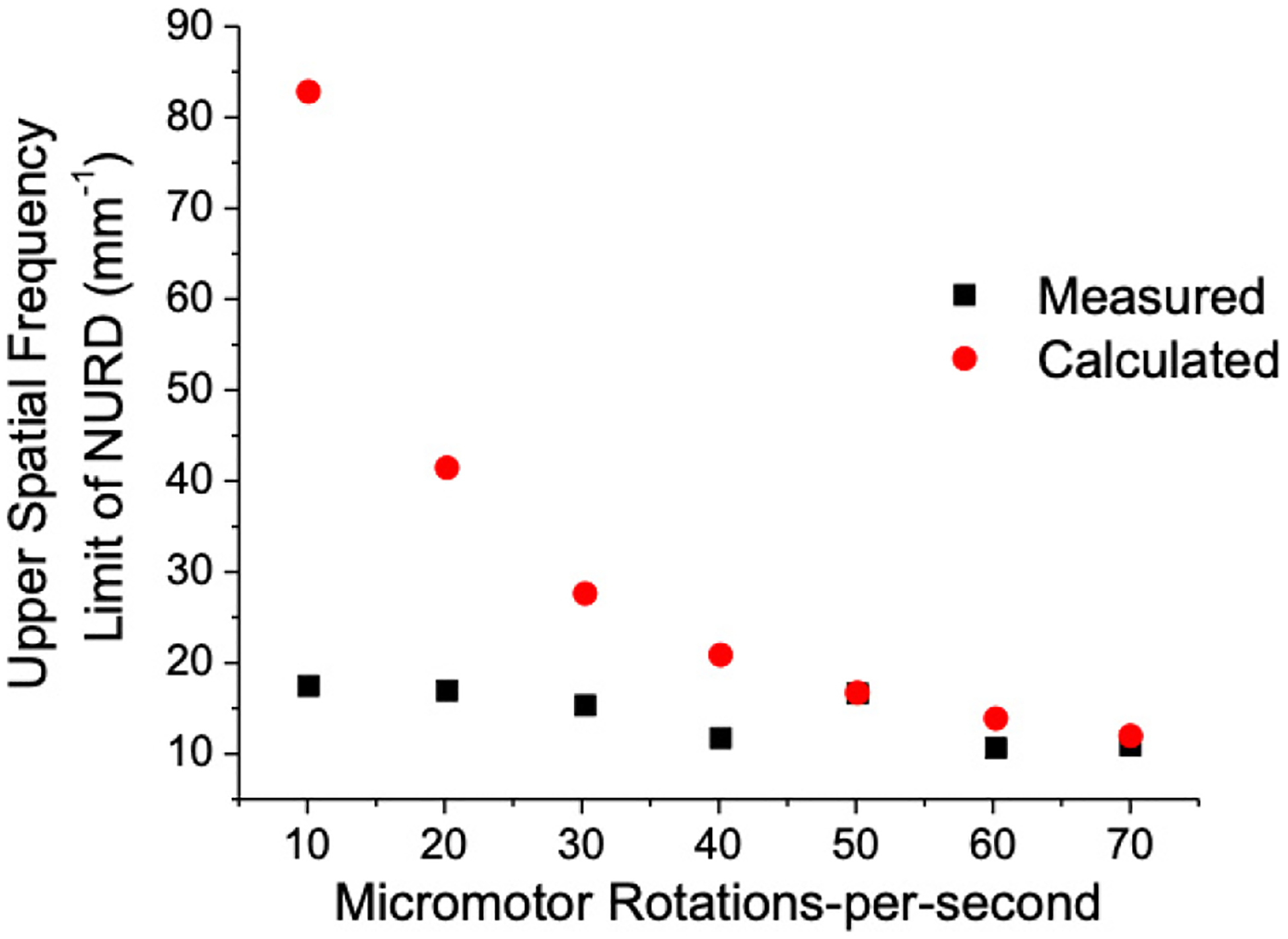
Upper spatial frequency limit of NURD regions versus rotational velocity of the micromotor. The red dots denote the spatial frequency corresponding to 15% of the maximum spatial frequency spectrum present in the OCT B-scan images. The black dots denote the measured spatial frequency upper limit corresponding to the shortest NURD region for a given rotational velocity. Each measured data point represents an average of the spatial frequency the shortest NURD regions identified in 20 OCT B-scans for a given rotational velocity.

In order to better characterize the NURD upper spatial frequency limit, we calculated the maximum spatial frequency for each image and compared 15% of this value to the measured NURD region for different frame rates. The red data points in Fig. 2 denote the spatial frequency that corresponds to 15% of the maximum spatial frequency in an OCT B-frame for a given rotational velocity. The maximum spatial frequency was calculated as mentioned above based upon the number of A-lines in the frame, *A*, and the circumference at the focal point of the endoscope, *C*. Here, it is assumed that the A-line rate is held constant at 70 k A-lines/s. Thus, 15% of the maximum spatial frequency would be calculated as $f_{15\%}=0.15\left(\frac{A}{2C}\right)$. This measurement showed that the shortest NURD cluster typically occupies the lowest 15% of the spatial frequency spectrum as shown in Fig. 2.

The black data points in Fig. 2 correspond to the spatial frequencies calculated from the shortest NURD regions measured in an OCT B-frame for a given rotational velocity. Each black data point is the average of the (NURD) spatial frequency over 20 OCT B-frames. We found that this curve was representative of several endoscopes manufactured with the same basic design, i.e., with the same model of micromotors and the same load of due to micro-mirrors.

**Fig. 3.**
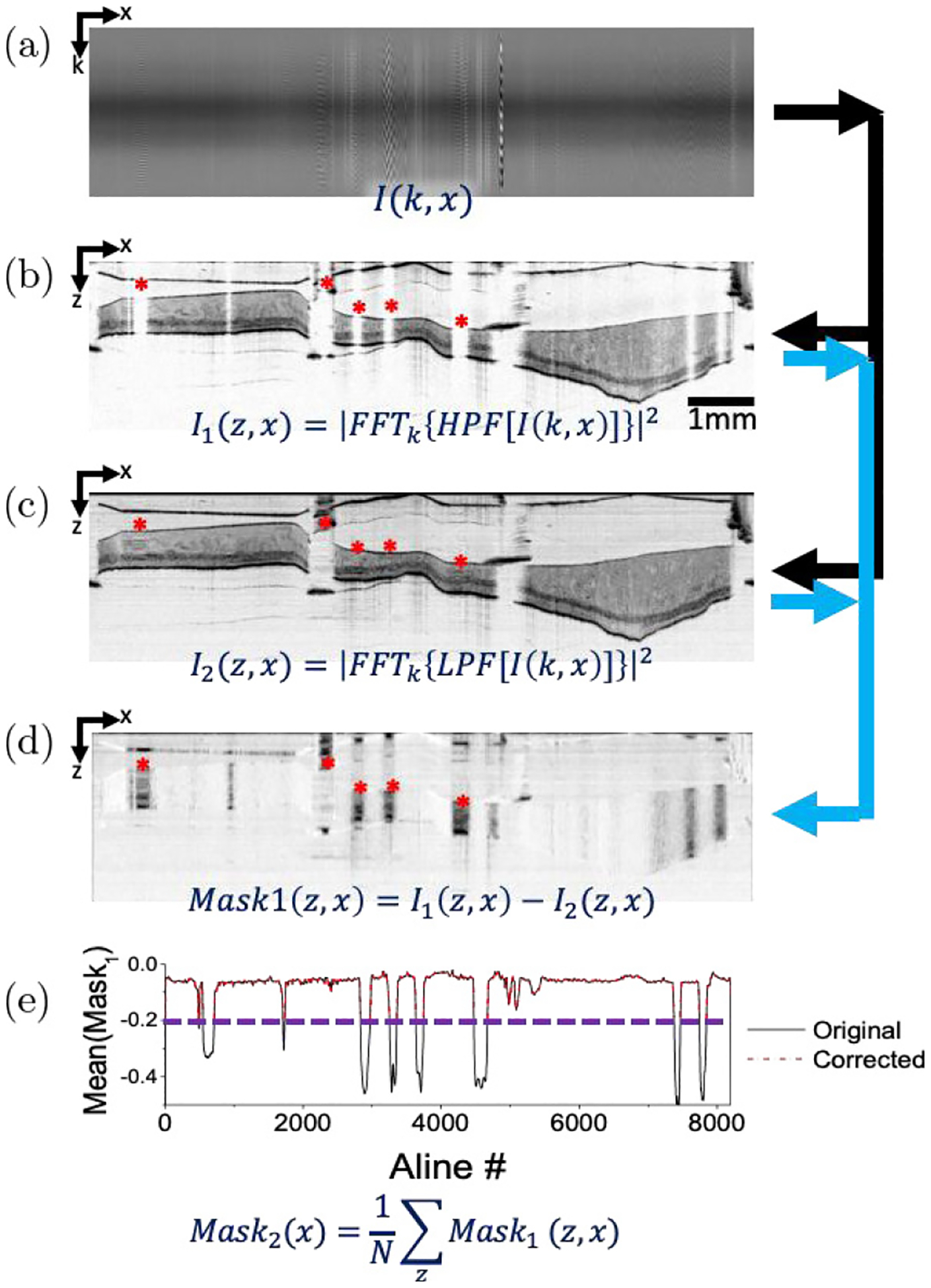
Flowchart representation of NURD correction algorithm. NURD sections are indicated by red asterisks. (a) The raw spectral interference fringes in one frame acquired by the SD-OCT system. (b) and (c) Images *I*_1_(*z*; *x*) and *I*_2_(*z*; *x*) resulting from high-pass and low-pass frequency filtering of the raw OCT image data, respectively, prior to performing Fourier transform. (d) Mask1 obtained after performing image subtraction of the high-pass and low-pass frequency filtered images (i.e. *Mask*_1_ = *I*_1_ − *I*_2_). (e) *Mask*_2_ (shown in black) is obtained from averaging every A-line along the imaging depth from *Mask*_1_. The purple dashed line is an example of an appropriate threshold level for this dataset and the overlaid red dashed-line is the result of thresholding.

This cutoff frequency determines how effectively NURD regions are filtered out and 15% is the minimum cutoff frequency that effectively removes all visible NURD regions. Figure 3(a) shows the raw OCT image data *I*(*k*, *x*) obtained from the endoscopic SD-OCT system and Fig. 3(b) shows the corresponding high pass filtered image i.e., *I*_1_(*z*, *x*) = |*FFT*_*k*_{*HPF*[*I*(*k*, *x*)]}|^2^, with signal void regions pertaining to NURD regions. While the cutoff frequency preserved the relevant image data and effectively filtered out NURD regions in the image, certain low spatial frequency components relevant to true tissue structure may also have been filtered out.

Hence, the second step in the algorithm is to consider how to minimize the loss of low spatial frequency components belonging to tissue structure. This is done by performing low pass filtering (LPF) on the raw OCT image data prior to performing the FFT, i.e., *I*_2_(*z*, *x*) = |*FFT*_*k*_{*LPF*[*I*(*k*, *x*)]}|^2^. The LPF cutoff frequency was empirically determined to be 85% of the spatial frequency range and this cutoff frequency smoothed the transitions between tissue structure and NURD regions, as seen in Fig. 3(c). Independently, *I*_1_ and *I*_2_ exclude parts of the frequency content relating to tissue structure; however, the difference of these two images, i.e., *Mask*_1_ = *I*_1_(*z*, *x*) − *I*_2_(*z*, *x*), forms an image that accurately identifies the NURD regions.

Finally, *Mask*_1_ is collapsed into a single array of values by averaging along the imaging depth, i.e., *Mask*_2_ = Σ_*z*_*Mask*_1_ (*z*, *x*)/*N*, where *N* is the total number of pixels in an A-line. It is noted that *Mask*_2_ will have a lower value for NURD regions than tissue regions considering the function of *Mask*_1_ was to remove most tissue-relevant structures except with NURD (of low spatial frequency) remained. As shown in Fig. 3(e), *Mask*_2_ (black line) has a small negative value (between 0 and −0.1) for most of the A-lines where NURD does not occur. The small negative value is due to the overall decrease in background noise that results from the high-pass filter when computing *I*_1_(*z*, *x*). In comparison, *Mask*_2_ in NURD regions have values less than −0.25. Thus, a threshold (i.e., −0.2) can be set and used [marked by the purple dashed line in Fig. 3(e)] to demarcate NURD regions. Thus, *Mask*_2_ essentially offers a map of the spatial locations of NURD regions for the original unfiltered OCT image. Finally, a corrected image is formed by rejecting A-lines at spatial location *x* (as identified in *Mask*_2_, by red dashed line overlay) where the value of *Mask*_2_ is less than the threshold. Thus, final image will comprise of A-lines that correspond to spatial location *x*, where the value of *Mask*_2_ is greater than the threshold. All images in the volumetric pull-back scan were corrected in the same fashion using the spatial locations derived from *Mask*_2_.

**Fig. 4.**
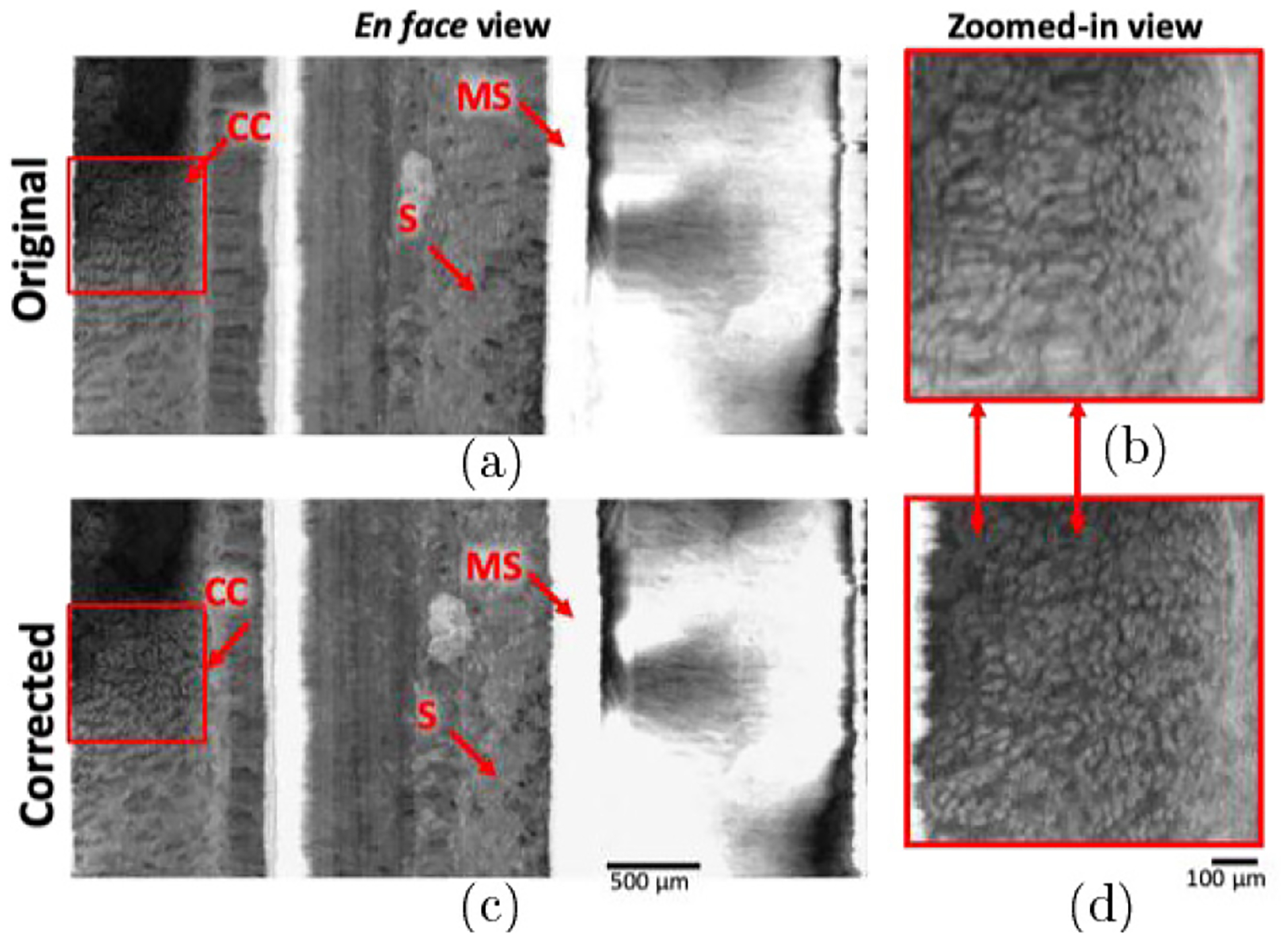
*En face* projection view of the 3D volumetric pull-back scan acquired from *ex vivo* rat colon. (a) Original pull-back scan with (b) zoomed-in region. (c) The NURD corrected pull-back scan and (d) corresponding zoomed-in region. The red arrows between the zoomed-in images in (b) and (d) indicate two NURD regions that are difficult to detect by eye and have been corrected with this algorithm.

Figures 4(a) and 4(c) show an example of original and corrected *ex vivo* images of rat colon, respectively. In order to better visualize NURD and the correction, the volumetric dataset was displayed as a flattened, rectangular projection view. Figures 4(b) and 4(d) on the right are the original and corrected zoomed-in regions of the area demarcated in red boxes. The two red arrows point out small sections of stick-slip NURD (which are more difficult to detect by eye) have also been identified and corrected, while the imaged tissue structure is preserved. Colonic crypts (cc) as well as remnants of stool (s) are visualized in the *en face* projection view.

## 3. Discussions and Conclusion

In this paper, a robust algorithm for correcting stick-slip NURD in endoscopic OCT images has been demonstrated. This algorithm utilizes the fact that stick-slip NURD results in clusters of repeated A-lines which can be identified in the low spatial frequency range of the raw OCT image data. Taking advantage of this property, a mask can be created to isolate NURD, remove unwanted A-lines, and reform the cross-sectional OCT image.

One of the benefits of this algorithm is that it creates a generic mask to be applied to an image with stick-slip NURD. In distal scanning systems, spatial positions of stick-slip NURD are specific to the micromotor (including its type and rotational velocity) and remain fixed with respect to the catheter enclosure (e.g., strut positions) throughout the pull-back scan and between datasets acquired with the same catheter and same imaging parameters (such as the rotational velocity) as long as the micromotor has not been stopped and restarted. Thus, comparing to correlation-based NURD correction algorithms in which correlations between adjacent regions (A-lines) have to be calculated not only for one single frame but also for the entire volumetric dataset, creating a mask is only a one-time cost, since it can be applied to all subsequently acquired datasets for a catheter with the same micromotor. At this junction, we would like to point out that NURD clusters differ in location and number of A-lines with varying rotational velocity. Thus, the proposed algorithm can be used to create reusable mask for each unique set of imaging parameters.

**Fig. 5.**
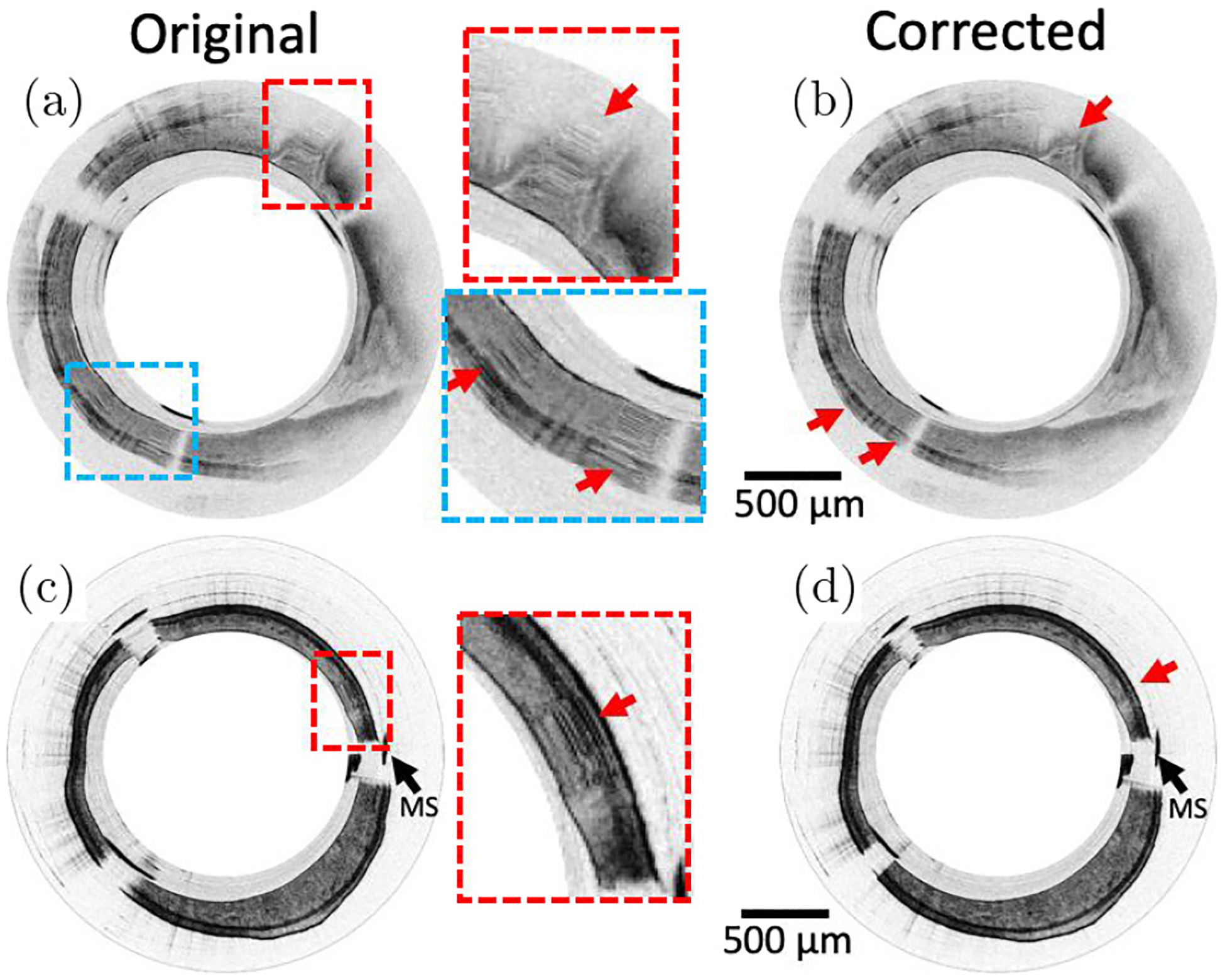
Original and NURD corrected cross-sectional OCT images from *in vivo* mouse colon (a) with zoomed-in NURD regions and (b) and *ex vivo* rat colon (c) with zoomed-in NURD regions and (d). Red arrows show NURD regions in the original images and locations of the NURD regions that have been removed by the generic mask-based algorithm.

Another benefit is that this algorithm does not require any fiducial markers. The lack of fiducial markers makes it easy to readily apply this algorithm to any dataset without modification of endoscope design. Figure 5 shows an example of how this algorithm can be easily adapted to catheters with different types of micromotors. The cross-sectional images shown in Figs. 5(a) and 5(b) are acquired from *in vivo* mouse colon using a distal scanning catheter with a 900 *μ*m diameter DC micromotor at 50 frames-per-second; Figs. 5(c) and 5(d) are acquired from *ex vivo* rat colon using the aforementioned 1.9 mm DC micromotor based catheter at 8 frames-per-second. With a one-time cost, a mask was created for each catheter using the generic algorithm.

This mask can now be applied to across subsequent frames within the dataset and across datasets acquired by the same catheter in the same run. By applying the algorithm in both *ex vivo* and *in vivo* scenarios, we can see that this algorithm is not influenced by bending of the catheter or any external pressure and it is purely based upon the performance (speed) of the micromotor.

While this algorithm is applicable to a range of scenarios in which stick-slip NURD occurs, the performance suffers greatly where stretch-shrink NURD (also known as motion blur) coexists with stick-slip NURD. Temporally, stick-slip NURD occurs when the rotational velocity changes are on time-scales significantly less than B-frame acquisition time. Spatially, it then manifests in localized regions of the imaging frame. Motion blur occurs when changes in the rotational velocity are on the same time-scale as B-frame acquisition affecting many A-lines or even the entire imaging frame. Spatially, this will contaminate the low spatial frequency ranges such that stick-slip NURD can no longer be isolated through spatial frequency filtering. In this case, the algorithm could only be applied after correction of motion blur.

In summation, this algorithm has shown its ability to correct NURD in distal scanning endoscopic OCT images. The algorithm is robust and can be applied to other endoscopic OCT images for correcting stick-slip NURD.
